# Optimizing locked nucleic acid/2’-O-methyl-RNA fluorescence *in situ* hybridization (LNA/2’OMe-FISH) procedure for bacterial detection

**DOI:** 10.1371/journal.pone.0217689

**Published:** 2019-05-31

**Authors:** Andreia S. Azevedo, Inês M. Sousa, Ricardo M. Fernandes, Nuno F. Azevedo, Carina Almeida

**Affiliations:** 1 CEB - Centre of Biological Engineering, University of Minho, Braga, Portugal; 2 LEPABE - Laboratory for Process Engineering, Environment, Biotechnology and Energy, Faculty of Engineering, University of Porto, Porto, Portugal; 3 i3S - Instituto de Investigação e Inovação em Saúde, Universidade do Porto, Porto, Portugal; 4 IPATIMUP - IPATIMUP, Institute of Molecular Pathology and Immunology of the University of Porto, Porto, Portugal; 5 INIAV, IP- National Institute for Agrarian and Veterinary Research, Rua dos Lagidos, Lugar da Madalena, Vairão, Vila do Conde, Portugal; Columbia University, UNITED STATES

## Abstract

Despite the successful application of LNA/2’OMe-FISH procedures for bacteria detection, there is a lack of knowledge on the properties that affect hybridization. Such information is crucial for the rational design of protocols. Hence, this work aimed to evaluate the effect of three essential factors on the LNA/2’OMe hybridization step—hybridization temperature, NaCl concentration and type and concentration of denaturant (formamide, ethylene carbonate and urea). This optimization was performed for 3 Gram-negative bacteria (*Escherichia coli*, *Pseudomonas aeruginosa* and *Citrobacter freundii*) and 2 Gram-positive bacteria (*Enterococcus faecalis* and *Staphylococcus epidermidis*), employing the response surface methodology and a Eubacteria probe. In general, it was observed that a high NaCl concentration is beneficial (from 2 M to 5 M), regardless of the denaturant used. Urea, formamide and ethylene carbonate are suitable denaturants for LNA/2’OMe-FISH applications; but urea provides higher fluorescence intensities among the different bacteria, especially for gram-positive bacteria and for *P*. *aeruginosa*. However, a unique optimal protocol was not found for all tested bacteria. Despite this, the results indicate that a hybridization solution with 2 M of urea and 4 M of NaCl would be a proper starting point. Furthermore, a hybridization temperature around 62°C, for 14 bp probes with LNA monomers at every third position of 2′OMe and 64% of GC content, should be use in initial optimization of new LNA/2’OMe-FISH protocols.

## Introduction

Fluorescence *in situ* hybridization (FISH) is one of the most well-established molecular biology techniques used for the rapid and direct detection, localization and quantification of microorganisms in many fields of microbiology (e.g. [1–8]). This technique is based on the hybridization of fluorescently-labeled molecules (also called probes) with the conserved 16S or 23S rRNA sequences, particularly abundant and relatively stable in viable cells [9].

During the last years, the application of nucleic acid mimics (NAMs) have emerged for *in vivo* and *in vitro* applications in the health area (e.g. [10–13]). In particular, locked nucleic acid (LNA) and 2’-O-methyl-RNA (2’OMe), have been adapted for FISH procedures (e.g. [4, 6]), to overcome some of the drawbacks that have been associated to DNA -FISH. For example, low cell permeability, low hybridization affinity, sensitivity to nucleases and low target site accessibility have been associated to DNA-FISH method [14–17]. In addition, both LNA and 2’OMe probes, offer higher design flexibility comparatively to the PNA and DNA probes [18]. As an example, in PNA probes the addition or removal of single nucleotides will cause a significant change in the molecule melting temperature, usually of around 4 °C. In opposition, with LNA/2’OMe probes fine-tuning is possible by intercalating LNA and 2’OMe monomers. Thus, minor adjustments in the melting temperature can be achieved by changing the type of nucleotide in that position. This would allow for a thorough control of the thermodynamic parameters (e.g. different probes with similar melting temperature), facilitating multiplex approaches (detection of multiple targets simultaneously) or detection of bacteria under specific conditions (e.g. at human body temperature [6, 7]). While the potential of such combinations is almost unlimited due to the number of new NAMs that are emerging; introducing LNA monomers at every third position of 2′OMe probes is a common approach used to improve FISH experiments in terms of affinity and sensitivity [18, 19].

Several studies have shown that LNA monomers can increase affinity toward DNA and RNA molecules, provide resistance to nuclease degradation, and improve signal-to-noise ratio, sensitivity and specificity [20–23]. Also, DNA duplexes containing LNA monomers present higher thermal stability and are able to increase the melting temperature, per single LNA nucleotide incorporation (1 °C to 8 °C against DNA and 2 °C to 10 °C against RNA) [23–25].

2′OMe is another RNA mimic, which displays a high nuclease resistant and a great affinity for RNA/DNA targets [26–28]. Despite the reported advantages of LNA/2’OMe molecules in terms of improving the accuracy, stability, robustness and simplicity of the FISH, they have not been systematically tested in microorganisms. This information is crucial for stablishing the true potential of these molecules, as for any other synthetic nucleic acid.

The hybridization step in FISH procedure is influenced by several factors including temperature, ionic strength and denaturing conditions [29]. Moreover, FISH efficiency also depends of others factors including ribosome content of cells, cell wall permeabilization, affinity of the probe, accessibility of the targets, type of fluorochromes coupled to the probe, and the type of equipment used to analyze the FISH signal [2, 30–32]. Hence, as different variables affect the FISH protocol, numerous studies have been working on its optimization (e.g.[3, 33–38]). However, in terms of NaCl concentration and type of denaturant, there is a lack of an established FISH protocol using LNA/2’OMe probes for bacteria detection individually or in multiplex assay (e.g. [4, 7, 39]). As such, in this study, the effect and the interplay of hybridization temperature, NaCl and denaturant (formamide, urea and ethylene carbonate) concentration on LNA/2’OMe-FISH was modulated using an universal *Eubacteria* LNA/2’OMe probe (EUB388) [1] through response surface methodology (RSM) [37, 38].

## Materials and methods

### Bacterial strains and growth conditions

The bacterial strains selected for this study were *Escherichia coli* CECT 515, *Pseudomonas aeruginosa* PAO1, *Citrobacter freundii* SGSC 5345, *Staphylococcus epidermidis* RP62A and *Enterococcus faecalis* CECT 184. All cultures were grown on tryptic soy agar (TSA, 3% (w/v) tryptic soy broth and 1.5% (w/v) agar) (Liofilchem Diagnostic, Italia), overnight at 37 °C.

### LNA/2’OMe probe design and synthesis

In order to evaluate the effect of hybridization temperature, type and concentration of denaturant (formamide, urea or ethylene carbonate) and NaCl concentration on the fluorescence intensity (a.u.) of LNA/2’OMe-FISH method, the hybridizations were performed in suspension based on Azevedo *et al*. [4], followed by signal quantification using flow cytometry. In order to identify all the bacteria in this study, a universal LNA/2’OMe probe EUB338 (5’- mT*lG*mC*mC*lT*mC*mC*lC*mG*mT*lA*mG*mG*lA*-3’) based on Amann *et al*. [40], specific for the *Eubacteria* domain, was used during the FISH experiments. LNAs (represented by “l”) were placed at every third 2’OMe (represented by “m”) monomer. The LNA/2’OMe probe EUB338 also included a phosphorothioate backbone (PS linkage represented by “*”) instead of a phosphodiester. The choice of this type of probe design was based on previous works of our group (e.g. [4, 7]), which demonstrated that the detection and identification of bacteria by FISH may benefit from the properties of the LNA/2’OMe probes with a PS linkage. The probe was synthesized and labelled at the 5’ end with FAM (Ribotask, Odense, Denmark).

### LNA/2’OMe-FISH method combined with flow cytometry analysis

First, the bacterial cells were harvested from TSA plates and suspended in sterile water to a final concentration of ~10^9^ cells.ml^-1^. Then, for sample fixation/permeabilization, cell suspensions were centrifuged (10 000 x g, 5 min) and fixed in 400 μL of 4% (w/v) paraformaldehyde (Sigma, USA) for 1 hour at room temperature. After centrifugation, the fixed cells were resuspended in 500 μL of 50% (v/v) ethanol and incubated at -20°C for at least 30 min. For hybridization, 100 μL of the fixed cells were pelleted by centrifugation (10 000 x g, 5 min) and resuspended in 100 μL of hybridization solution containing 200 nM of LNA/2’OMe probe EUB338, 50 mM Tris-HCl (pH 7.5) (Sigma, USA), denaturant (formamide [Sigma, USA], urea [National Diagnostics, USA] or ethylene carbonate [Sigma Aldrich, Garman]) and NaCl (Fisher Scientific, Belgium). Samples were incubated during 60 min at the hybridization temperatures under study. Afterwards, samples were centrifuged, resuspended in 500 μL of washing solution containing 5 mM Tris-base (pH 10; Sigma, USA), 15 mM NaCl (Fisher Scientific, Belgium) and 0.1% (v/v) Triton X-100 (Sigma, USA) and incubated for 30 min at the same temperature used in the hybridization step. Finally, the suspension was pelleted by centrifugation (10 000 g for 5 min) and resuspended in 500 μL of 0.9% (w/v) NaCl (Fisher Scientific, Belgium). Each experiment was performed in triplicate. Negative controls were resuspended in hybridization solution with no probe. The samples were stored at 4 °C in the dark before flow cytometry analysis.

The flow cytometry analysis was performed to evaluate the fluorescence intensity of hybridized samples and negative controls. For this, a Sony Biotechnology EC800 (Sony Biotechnology Inc., Champaign, IL, USA) equipped with a 488 nm argon ion laser was used. Forward angle light scatter (FS), side angle light scatter (SS), and green (FL1) fluorescence were detected at logarithmic scale. A minimum of 40 000 events falling into the bacterial gate defined on the FSSS plot were acquired per sample. The data were analyzed with the EC800 software version 1.3.6. (Sony Biotechnology Inc., Champaign, IL, USA), and the average fluorescence intensity was determined for each triplicate experiment.

### Response surface methodology (RSM) and statistical analysis

RSM, a mathematical and statistical tool based on the fit of empirical models to the experimental data obtained in relation to experimental design [41], was employed to study and optimize the hybridization efficiency of the LNA/2’OMe EUB338 probe in bacteria. The average fluorescence intensity obtained for each sample, after LNA/2’OMe-FISH, was used as the dependent variable. Hence, RSM tool was employed according to the procedure applied by previous works of our group [37, 38, 42]. The standard central composite designs (CCD) were set up for *E*. *coli* CECT 515, *P*. *aeruginosa* PAO1, *C*. *freundii* SGSC 5345, *S*. *epidermidis* RP61A and *E*. *faecalis* CECT 184, using the statistical software Design Expert 11 (Stat-Ease Inc., Minneapolis, USA) to estimate the coefficients of the model. For the designs, the hybridization temperature (variable *x1*), denaturant concentration (*x2*) and salt concentration (*x3*) were considered the independent variables and the fluorescence intensity was the response. Each of the three variables assumed five different experimental values, defined according our previous studies and the results obtained within this study (Table 1 and S1, S2 and S3 Tables). Each CCD included 2^3^ factorial points (coded as ±1), 6 axial points (coded as ± α) that represent extreme values used for the estimation of the model curvature, and a center point (all factors at coded level 0) repeated 6 times to take into account the experimental error [43]. The design matrix consisted of 20 LNA/2’OMe-FISH experiments and each experiment was performed in triplicate.

**Table 1:** Experimental levels for the variables (hybridization temperature, NaCl and denaturant) defined in the first optimization assays of the LNA/2’OMe-FISH hybridization protocol for *E*. *coli*, *P*. *aeruginosa*, *C*. *freundii*, *S*. *epidermidis* and *E*. *faecalis*.

Assay	Variables	Range and level				
		-α	-1	0	+1	+α
**1^a^**	x_1_ Hybridization temperature (°C)	40.0	50.1	65.0	79.9	90.0
	x_2_ [NaCl] (M)	1.50	2.41	3.75	5.09	6.00
	x_3_ [Formamide] (% V/V)	0.00	3.55	8.75	13.95	17.50
**1^a^**	x_1_ Hybridization temperature (°C)	40.0	50.1	65.0	79.9	90.0
	x_2_ [NaCl] (M)	1.50	2.41	3.75	5.09	6.00
	x_3_ [Ethylene carbonate] (% V/V)	0.00	3.55	8.75	13.95	17.50
**1^a^**	x_1_ Hybridization temperature (°C)	40.0	50.1	65.0	79.9	90.0
	x_2_ [NaCl] (M)	1.50	2.41	3.75	5.09	6.00
	x_3_ [Urea] (% P/V)	0.50	1.41	2.75	4.09	5.00

^1a^ Other assays were performed when the experimental levels were not appropriate according to the model.

To find the optimum hybridization conditions of the five species, the obtained fluorescence values were fitted to a quadratic model. Each model was analyzed using analysis of variance (ANOVA) to test the significance and adequacy of the model. The Fisher variance ratio (Model F-value) was also evaluated. Model F-value is a measurement of variance of data about the mean based on the ratio of mean square of group variance due to errors; high Model F-value indicates that implies the model is significant.

Finally, the optimum conditions within the experimental range that maximize the fluorescence intensity were estimated using the optimization function of the Design Expert software. The value estimated for the optimum conditions was, then verified for each bacterium on a confirmation experiment, in triplicate.

### Evaluation of the *E*. *coli* LNA/2’OMe probe specificity

To evaluate whether each bacterium can be detected with accuracy under optimum conditions, an *E*. *coli*-specific LNA/2’OMe probe (5’-FAM-lC*mA*mC*lG*mC*mC*lT*mC*mA*lG*mC*mC*lT*mU*mG*lA*-3’) was tested against all 5 bacteria. The hybridizations were performed at the optimal hybridization conditions for *E*. *coli* as described above. Afterwards, 20 μl of the suspension was spread on a microscope slide which was allowed to air dry before microscopy visualization. Negative controls were included.

Images were acquired using a Leica DM LB2 epifluorescence microscope (Leica Microsystems GmbH, Wetzlar, Germany) connected with Leica DFC300 FX camera (Leica Microsystems GmbHy, Germany) and equipped with a sensitive filter to the FAM fluorochrome (BP 450–490, FT 510, LP 515 for). The images were captured using a Leica IM50 Image Manager with a magnification of x1000, and fluorescence intensities were quantified by using an open source image-processing ImageJ software [7]. Data was plotted as mean of arbitrary fluorescence units (a.u) which represented the mean fluorescence intensity.

## Results and discussion

Despite the successful applications of LNA/2′OMe-FISH in bacterial identification (e.g. [4, 44, 45]), there is an absence of studies that assess the impact of denaturant and salt concentration on its efficiency. Hybridization temperature was also modeled in order to assess an optimal range for a standard LNA/2′OMe-probe design (probes with ~14 nucleotides, 64% of GC content, one LNA at very third position of the 2′OMe and phosphorothioate backbones). However, the size and number of residues are aspects that will have great impact on the probe temperature range. This information is very important to find the more suitable hybridization conditions for bacteria detection and to move towards a tailored design of hybridization experiments.

Due to the large number of variables involved in a FISH method (e.g. temperature and time of hybridization, probe concentration, type of microbial cells targeted, type and concentration of denaturing agents, salt concentration), optimization is normally performed using a trial-and-error approach. We are currently using response surface methodology (RSM), a statistical approach that allows to obtain an optimum in a cost-effective way. Using the RSM the number of experiments that need to be performed is significantly reduced, allowing us to test a large number of variables and better understand the relationship between them.

RSM was applied to model the data obtained from 3 Gram-negative (*E*. *coli*, *C*. *freundii* and *P*. *aeruginosa*) and 2 Gram-positive species (*E*. *faecalis* and *S*. *epidermidis*). Different genera were selected to include bacteria with different characteristics, including different cell wall composition. Furthermore, three denaturant agents were included, because even though the formamide is more frequently used in FISH methodology, there are also studies using less hazardous compounds as denaturant agents, including urea (e.g. [4, 7]) and ethylene carbonate (e.g. [46]).

### Study design with response surface methodology

For each denaturant, an initial CCD was designed based on the values typically described in the literature (Table 1—initial conditions tested) and tested in Gram-negative and -positive bacteria. When an optimum value from response surface plots was not obtained, the CCD was redesigned (S1 Table—assay 2–5; S2 Table—assay 2–3 and S3 Table—assay 2–4). Using those designs, most of the quadratic models obtained for each bacterium were highly significant, as is evident from high Model F-values and low *p*-values (*p*<0.05) (S4 Table), confirming the adequacy of the model fits. Furthermore, the coefficients of determination, R^2^, confirmed a good fit between predicted values and the experimental data points.

Hence, using the successful modelling of the hybridization temperature, NaCl and denaturant concentration, we have been able to obtain the optimum hybridization conditions that lead to the maximum intensity fluorescence for all bacteria (Table 2). In general, experimental fluorescence values obtained in the confirmation experiments agree with the predicted fluorescence maximum values (Table 2).

**Table 2:** Optimal ranges of hybridization temperature, NaCl and denaturant concentration predicted through the RSM models for the tested bacteria. The predicted and experimental fluorescence values are also shown. Optimum ranges for each parameter have been stablished assuming a fluorescence intensity of at least 85% of the maximum value.

Denaturant	Bacteria	Hybridization conditions			Predicted fluorescence (a.u.)	Obtained fluorescence (a.u.)*
		Temperature (°C)	[NaCl] (M)	[Denaturant] (M; % v/v)		
**Formamide**	*E*. *coli*	51.4–76.1	0.03–1.99	1.18–4.40	296.13–348.87	369.14 ±32.62
	*P*. *aeruginosa*	56.4–74.2	2.11–4.92	4.27–12.80	322.97–379.97	225.30 ± 14.58
	*C*. *freundii*	50.0–79.0	2.25–5.00	9.00–17.69	113.76–133.83	141.26 ± 38.86
	*E*. *faecalis*	50.0–69.3	2.66–5.00	22.00–32.81	288.31–339.19	131.15 ± 4.31
	*S*. *epidermidis*	62.0–82.0	2.00–5.00	3.00–13.00	47.51–55.89	296.62 ± 46.55
**Ethylene carbonate**	*E*. *coli*	55.5–77.9	2.00–5.00	3.13–12.42	278.02–327.08	346.03 ± 19.93
	*P*. *aeruginosa*	53.8–78.1	2.03–3.42	4.02–12.71	196.01–230.61	291.57 ± 14.15
	*C*. *freundii*	57.9–79.0	2.00–4.89	3.76–13.00	266.56–313.59	353.14 ± 12.17
	*E*. *faecalis*	46.0–63.0	2.00–5.00	1.00–4.00	174.27–205.03	126.34 ± 20.76
	*S*. *epidermidis*	62.0–82.0	2.90–5.00	0.00–2.00	106.68–125.50	254.37 ± 17.45
**Urea**	*E*. *coli*	50.1–60.0	2.41–5.01	1.41–4.09	189.217–236.52	253.87 ± 13.73
	*P*. *aeruginosa*	47.1–58.1	2.45–5.01	0.67–2.37	395.90–465.75	324.35 ± 18.26
	*C*. *freundii*	50.2–63.3	2.42–4.57	0.63–2.08	183.51–215.89	225.82 ± 39.74
	*E*. *faecalis*	50.1–79.9	2.59–5.01	1.41–4.09	345.03–405.92	205.58 ± 17.75
	*S*. *epidermidis*	64.2–82.9	3.37–5.01	0.61–1.84	188.04–221.22	314.17 ± 21.52

*The obtained fluorescence was evaluated using the optimum hybridization temperature, denaturant and salt concentration predicted through the RSM models for each bacterium.

Recent studies on PNA-FISH optimization, have noticed that Gram-negative bacteria show higher fluorescence intensity signals compared with Gram-positive bacteria [37, 38]. This has been mainly attributed to difficulties in permeabilization (e.g. *S*. *epidermidis*, *Listeria innocua*, *Bacillus subtilis*) and, thus, low fluorescence intensities in FISH procedure could be observed [37, 38, 47]. In the present study, a slight variation among the species is noticed; but a correlation with the gram-type is not clear. However, according Carr *et al*. [48], explaining the intensity fluorescence based on cell permeability properties is insufficient; in fact, to compare the fluorescence intensity among species, the cell size should also be taken into account. Nonetheless, in our study, the cells were collected at the same growth stage (exponential phase) for the optimization assays for each species. In this case, it would be unexpected that cell size has a great effect on the model obtained for each species. In addition, LNA/2′OMe probes have a shorter size that traditional DNA probes, which might help on the cell wall penetration on both Gram-types. However, as mentioned above, PNA probes diffusion in Gram-positives also seem to be somehow affected despite being also shorter than DNA and uncharged. While the size might help on crossing this barrier, some other unidentified factors should be involved.

In order to better understand the effect of the studied variables, the next section will discuss and explore the results in more detail for variables separately. Moreover, optimum ranges presented at the surface response plots (S1 and S2 Figs) will be analyzed for providing guidelines on balanced conditions for each variable (Table 2), when multiple species are to be targeted simultaneously. These optimum ranges have been established when the fluorescence intensity corresponded to at least 85% of the maximum value.

### Effect of salt concentration

**Fig 1:**
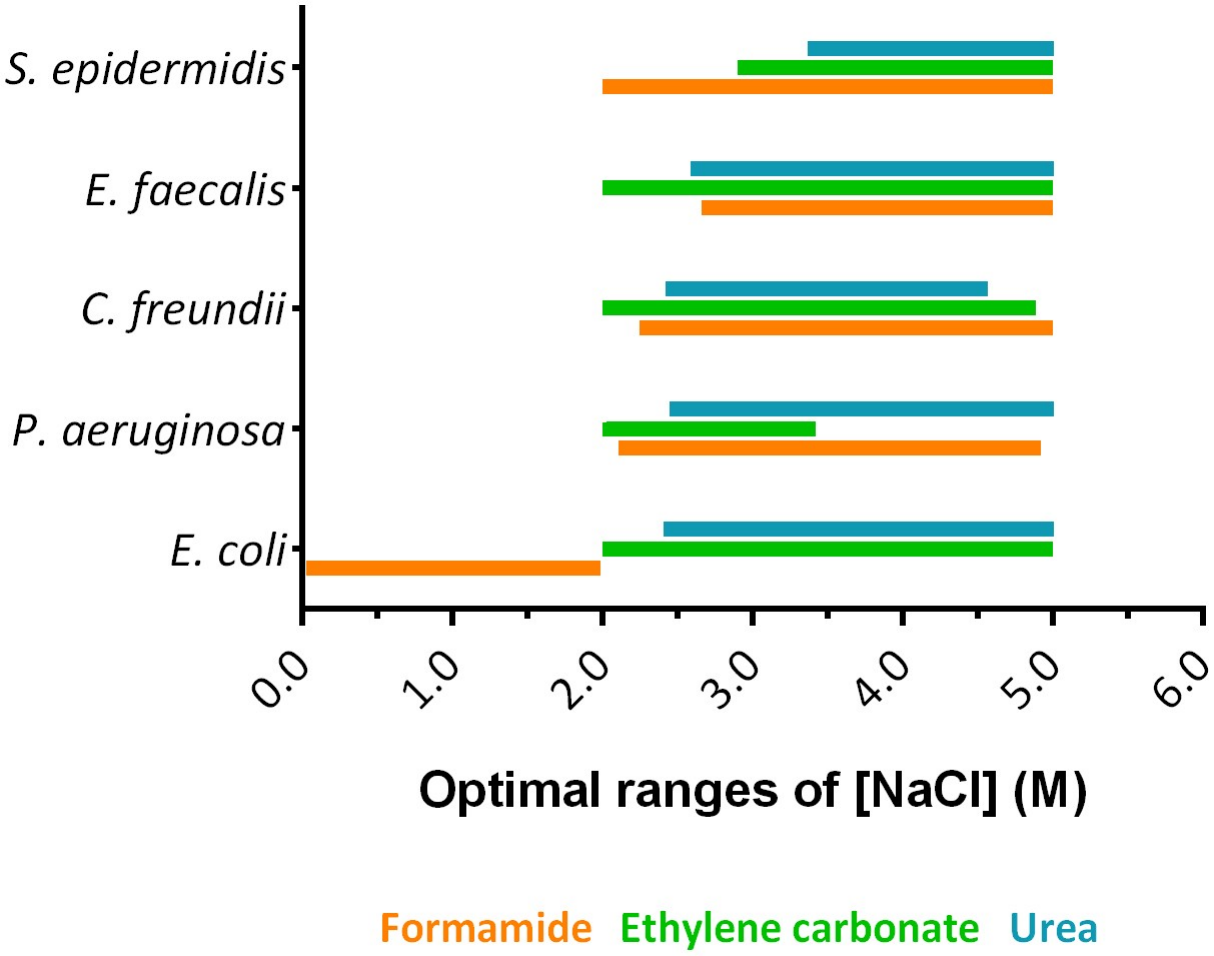
Representation of optimal ranges of NaCl concentration predicted through the response surface methodology models for the tested bacteria. Optimal ranges have been stablished assuming a fluorescence intensity of at least 85% of the maximum value.

Concerning ionic strength, supplementation with monovalent cations (e.g., sodium ions) to the FISH hybridization buffer have an important effect on thermodynamic stability [49, 50]. Adjustments on the sodium concentration in FISH protocols are needed, depending of the charge of NAMs being used. For instance, PNA is neutrally-charged, so low concentration of ions can be used as they are less relevant on the duplex stabilization [29]. For other NAMs, ions have a major role. Arora *et al*. [51] showed that high salt concentrations (Na^+^ and Mg^2+^) increased the stability of DNA probes containing LNA monomers. For LNA/2’OMe-FISH there are no studies that analyze the effect of NaCl concentration. In fact, NaCl is highly important in the hybridization to stabilize the repulsive interactions of LNA/2’OMe-rRNA duplexes. Hence, to understand the effect of NaCl concentration on LNA/2’OMe-FISH and select an optimum range of concentration (Fig 1), we plotted the NaCl concentration *versus* hybridization temperature, at the optimum denaturant concentration (S1 and S2 Figs). As it can be observed in Fig 1, those optimal values were not related with the type of denaturant. Surprisingly, the optimal NaCl concentration, ranging from ~2.0 M to ~5.0 M, is higher than those used in conventional LNA/2′OMe-FISH protocols (0.9 M) (e.g. [4, 6, 7]), except for *E*. *coli* when formamide was used (0.0–2.0 M). It is also important to note that PNA-FISH protocols have not described the same effect (e.g. [37, 38]); which might in fact be an important advantage of PNA, as it hybridization seems less susceptible to salt variations. The behavior here described could be related with the charged backbone of LNA and 2’OMe (the PNA backbone is uncharged). An appropriate salt concentration is essential to overcome electrostatic repulsion forces between negatively charged phosphates backbones of LNA/2’OMe probes and target. In addition, as the fluorescence intensity is very high when *E*. *coli* was treated with formamide, we believe that a higher NaCl concentration can also be used even if the intensity fluorescence is slightly affected.

### Effect of hybridization temperature

The optimum range hybridization temperatures is quite large, varying from ~50 °C to ~80 °C (Table 2, S1 and S2 Figs), regardless the denaturant being used. In fact, hybridization temperatures in the range of 55°C to 65°C are usually selected for NAM-FISH protocols (e.g. [3–5]), aiming at a compromise between sensitivity and specificity of the probe-target annealing reaction.

**Fig 2:**
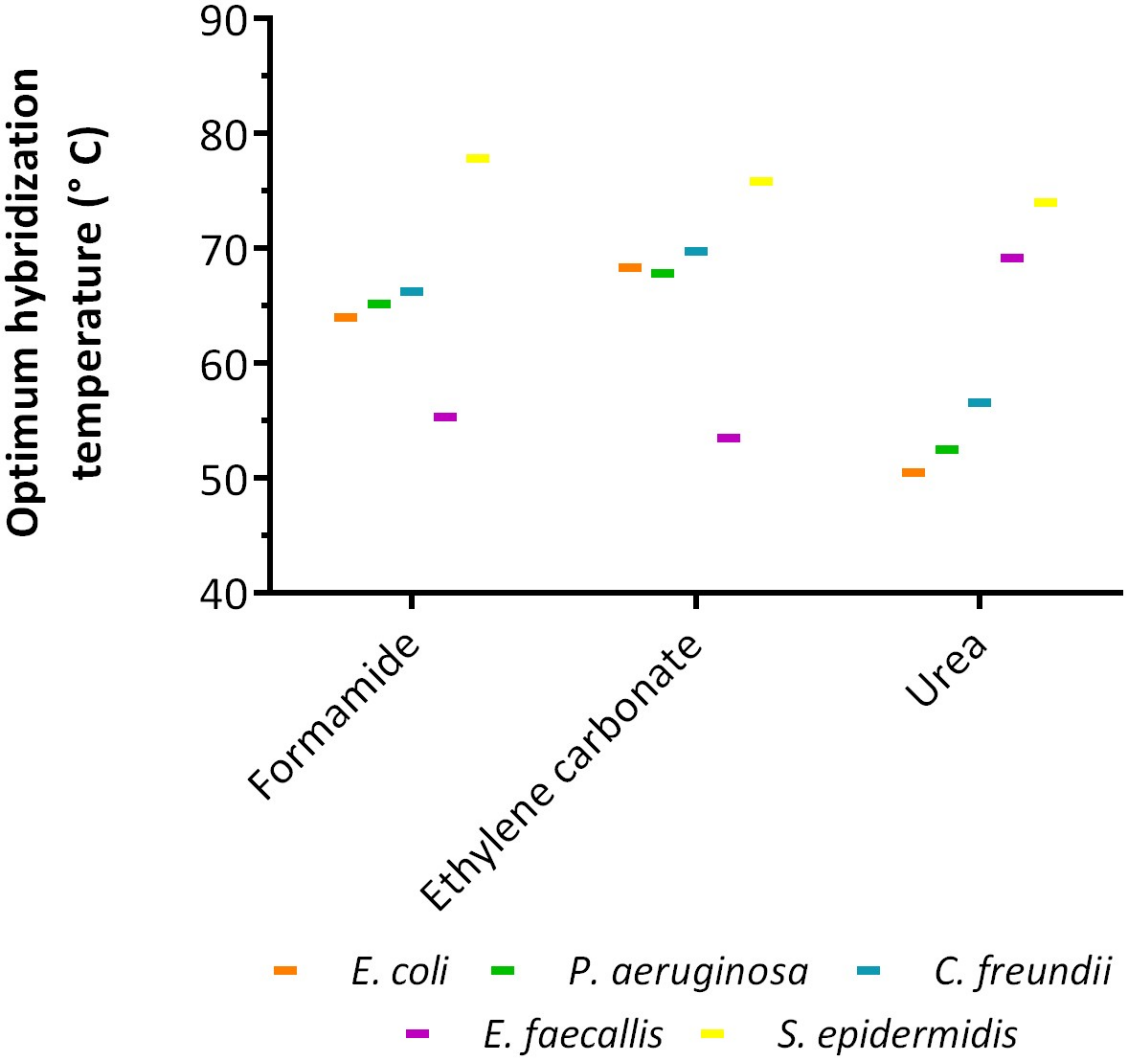
The optimum hybridization temperatures predicted through response surface methodology using a universal Eubacteria LNA/2’OMe probe (EUB388). Three denaturants were tested (formamide, ethylene carbonate and urea) during LNA/2’OMe protocol for detection of *E*. *coli*, *P*. *aeruginosa*, *C*. *freundii*, *E*. *faecalis* and *S*. *epidermidis*.

While it is expected that LNA/2’OMe probes with similar size and % GC content can work on this temperature range, it is important to bear in mind that this parameter is highly dependent on the sequence. Using the same sequence of LNA/2’OMe probe for the identification of all bacteria, it is expected to obtain the same optimum hybridization temperature. Surprisingly, as we can see in Fig 2, this behavior was not observed, which might indicate this is not a common feature of all bacteria, but instead might be species-specific.

In another study with PNA-FISH, Santos *et al*. [38] have demonstrated the same behavior. In fact, Tang *et al*. [52] reported that higher temperatures in the FISH protocol can increase the accessibility of the targeted rRNA to oligonucleotide probes and increase the permeability of cells by changing the structure of cell walls or membranes. Hence, the temperature used during the FISH protocol might affects each bacterium differently, since bacteria have very different properties in terms of cell size and cell wall or membrane structure. If the diffusion of the LNA/2’OMe probe through the cell and membrane wall is greatly affected by the temperature, it is expected that the optimal temperature of the FISH process will not the same for different bacteria.

### Effect of denaturant

**Fig 3:**
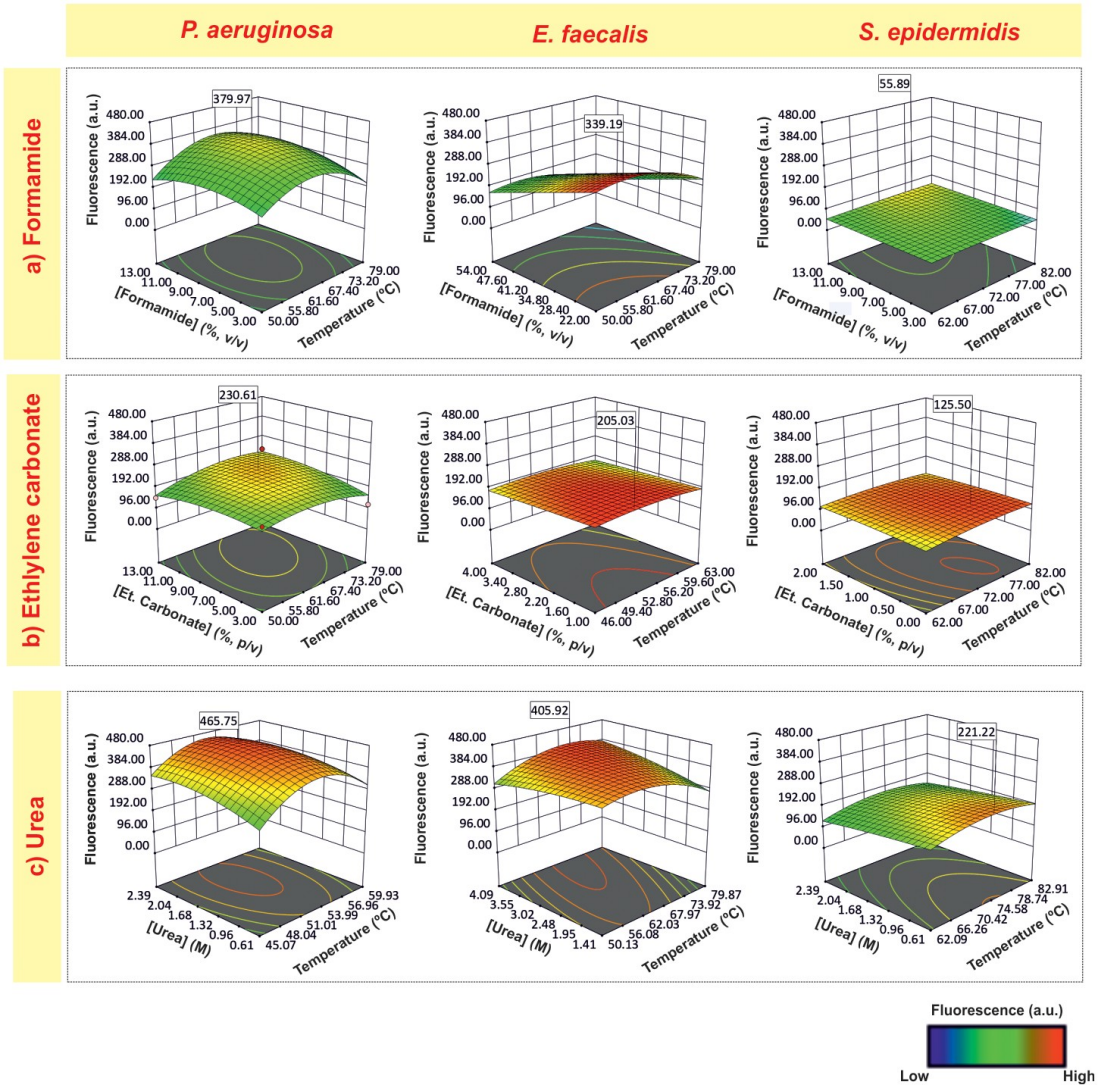
Surface response plots representing the interactions effect of hybridization temperature and formamide (a), ethylene carbonate (b) and urea (c) concentration on the fluorescence response of *P*. *aeruginosa*, *E*. *faecalis* and *S*. *epidermidis*. The NaCl concentration was kept constant at the optimum value for each bacterium. Fluorescence values are presented in arbitrary units (a.u).

To analyze the effect of denaturants, we plotted hybridization temperature *versus* denaturant concentration, with the NaCl concentration at the optimal value for each bacterium (Fig 3).

Of the 3 denaturants tested, formamide is the most widely used in FISH procedures for its thermodynamic effects on duplexes stability, allowing to perform the hybridization at lower temperatures [53]. Typically, in FISH methods, hybridization solution consists of 30–50% v/v formamide (e.g.[3, 7]); however, in the present study the optimum concentrations of formamide are quite lower (< 22% v/v) (Table 2). In fact, some authors have reported that low formamide concentrations could favor fluorescence intensity for other molecules[32, 38, 54–56]. In particular, Santos *et al*. [38] have reported that high concentrations of formamide could have a harmful effect on cell integrity, impairing the hybridization process.

Recently, the formamide replacement by other less hazardous chemicals, including urea and ethylene carbonate in the FISH procedure (e.g. [4, 46, 53]) has been suggested. For ethylene carbonate, the results indicated that higher ethylene carbonate concentrations (7.3–9.6% v/v) were a common feature for all Gram-negative bacteria; and, for Gram-positive bacteria, the range of ethylene carbonate concentration is 1.4–0.5% v/v) (Table 2).

**Fig 4:**
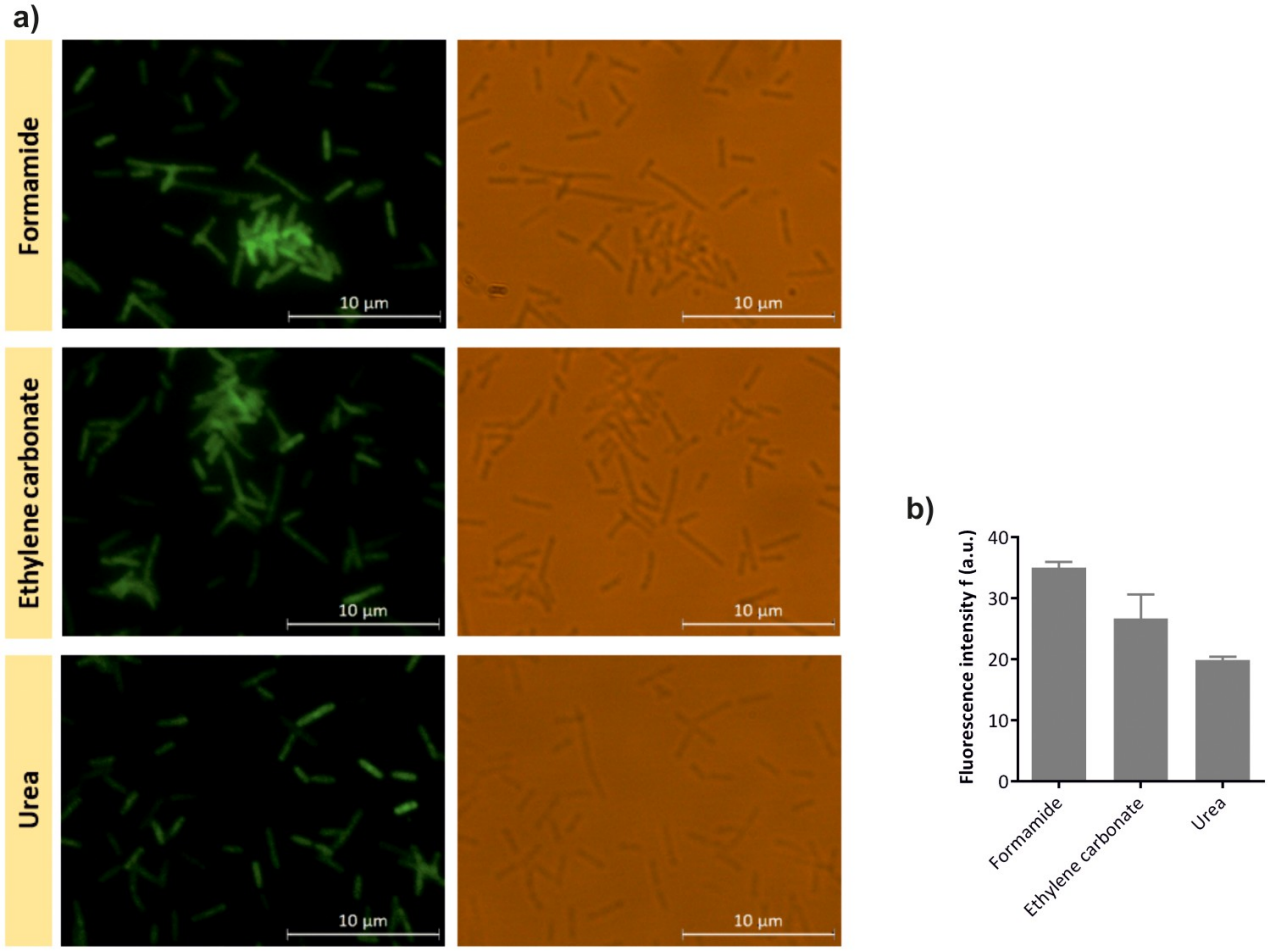
Evaluation of the *E*. *coli* LNA/2’OMe probe by epifluorescence microscopy. Epifluorescence and brightfield microscopy images of *E*. *coli*-specific LNA/2’OMe probe tested against *E*. *coli* at the optimal hybridization conditions (a). Fluorescence intensity of *E*. *coli*-specific LNA/2’OMe probe obtained by microscopy analysis; fluorescent signal intensity, expressed in arbitrary fluorescence units (a.u.), was evaluated using ImageJ software. Error bars represent standard deviation (b).

Concerning urea, previous studies have also shown its ability to substitute formamide in LNA-FISH methodology yielding an equally specific assay with high fluorescence intensity [4, 19, 39, 57]. Furthermore, the use of urea in LNA/2’OMe-FISH has provided higher fluorescence intensities [6]. In the present study, the hybridization solutions with urea seem to lead to higher intensity fluorescence compared with formamide and ethylene carbonate hybridization solutions, especially for gram-positive bacteria and for *P*. *aeruginosa* (Fig 3 and Table 2). Urea is a chaotropic agent that has also been studied for its effect on the permeabilization of cells [58, 59] and destabilization of proteins and nucleic acids [60, 61], which might enable a higher accessibility of the probe to the target. Taking the information on Table 2, another main observation is related with the fact that, when urea was applied to the LNA/2’OMe-FISH protocol, the ranges of the optimal urea concentration are overlapped (e.g. 1 M to 4 M of urea for *E*. *coli* and *E*. *faecalis*; 0.6 M to 2 M of urea for *P*. *aeruginosa*, C. *freundii* and *S*. *epidermidis*) (Fig 4a), which might simply the detection of several bacteria in a single experiment.

### Evaluation of the *E*. *coli* LNA/2’OMe probe specificity

**Fig 5:**
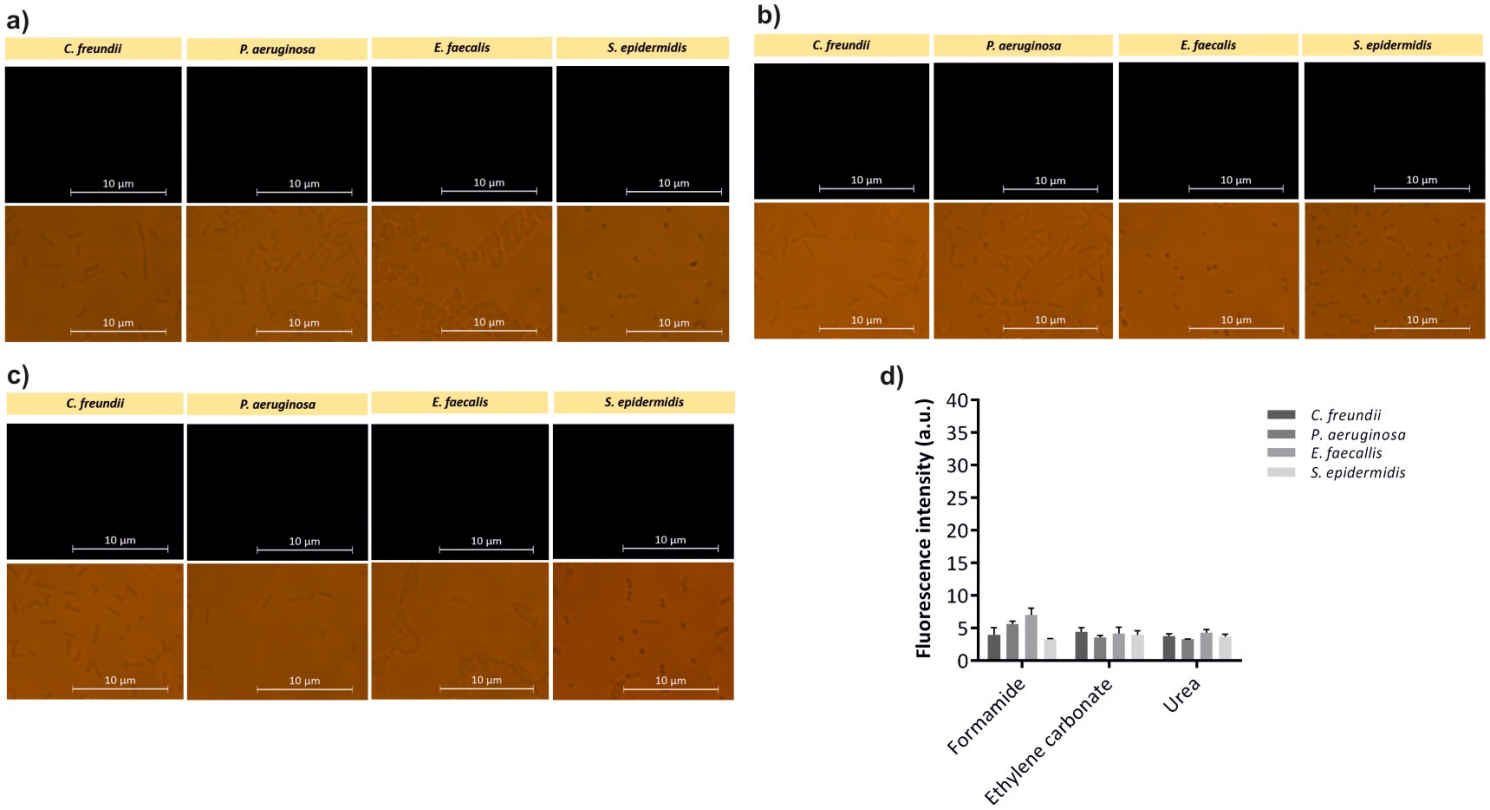
Assessment of the *E*. *coli* LNA/2’OMe probe specificity by epifluorescence microscopy. Epifluorescence and brightfield microscopy images of *E*. *coli*-specific LNA/2’OMe probe tested against non-target species using formamide (a), ethylene carbonate (b) and urea (c), as denaturing agents in the hybridization solution (a). Evaluation of the fluorescence intensity of *E*. *coli*-specific LNA/2’OMe probe obtained by microscopy, analysis using ImageJ software. Fluorescent signal intensity is expressed in arbitrary fluorescent units (a.u.). Error bars represent standard deviation (d).

The universal EUB338 probe is a useful model probe to understand the interplay of FISH variables in the optimal detection of different bacteria, with no influence of the target and probe sequence. However, whether these conditions can provide a specific detection is unclear, as optimization takes only into account the maximum signal. As high fluorescence intensity can arise from non-specific binding of the probe, the evaluation of the specificity of the probes is an important factor for the success of a FISH method. Hence, an *E*. *coli*-specific LNA/2’OMe probe was tested against the non-target species enrolled in this study. The probe was tested against all 5 bacteria using the optimal hybridization conditions obtained for *E*. *coli*. *E*. *coli*-specific LNA/2’OMe showed hybridization with *E*. *coli* CECT 434, and no cross-hybridization was observed to other species (Figs 4 and 5). As demonstrated in Fig 4a, it is possible to detect *E*. *coli* with accuracy at the optimal hybridization conditions. According the results presented in Fig 4b, the *E*. *coli*-specific LNA/2’OMe presents higher fluorescence intensity when formamide was used.

Overall, the results showed that *E*. *coli*-specific LNA/2’OMe probe was specific at optimal hybridization conditions for *E*. *coli* regardless of denaturing agent used. Nonetheless, even given an optimal hybridization protocol for a particular probe/species, each probe should always be evaluated against a panel of close microorganism are minor adjustments are likely to be needed.

## Conclusions

This work follows a set of optimization works that our research group has developed (e.g. [37, 38, 42]) to try to understand the parameters that are essential for the hybridization kinetics of nucleic acid mimics; so that we can move from a trial and error approach to a systematic optimization. While this study can provide a guide for further optimizations and help on establishing new LNA/2’OMe-FISH protocols, the optimal hybridization conditions will depend on the researchers’ goal. For instance, if a multiplex is expected, the conditions should be moved toward the species with weak signal to balance the overall outcome.

In general, the results showed that an optimized and standardized efficient LNA/2’OMe-FISH procedure is possible for bacterial detection, even when bacteria with different properties are present.

**Fig 6:**
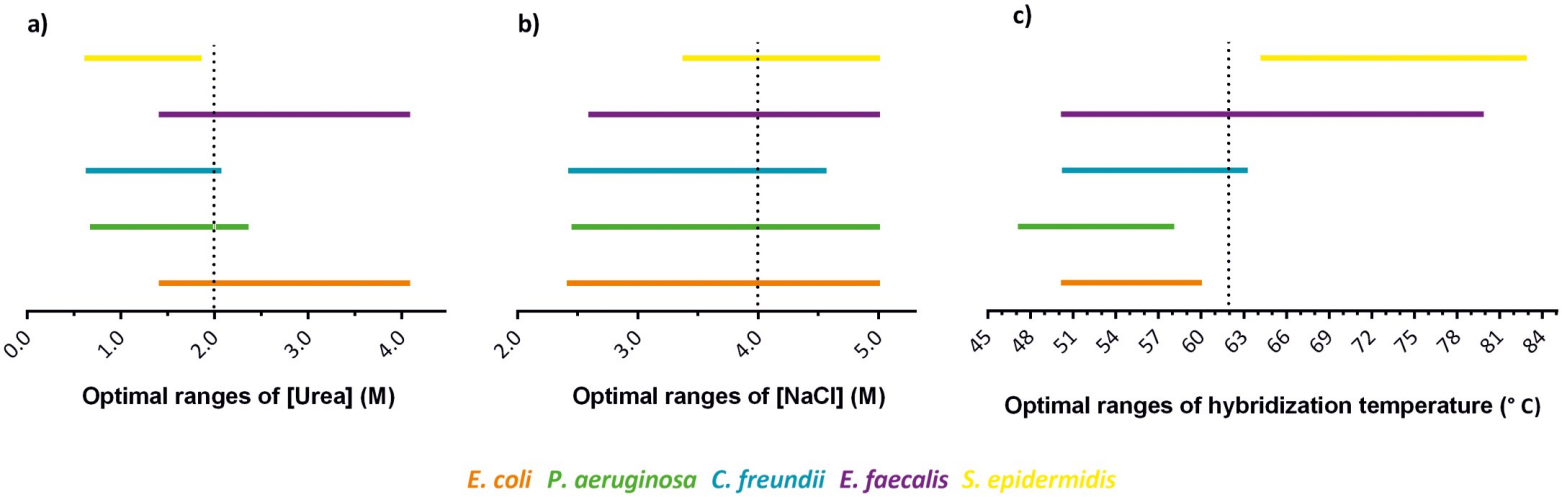
Representation of the optimal ranges of urea concentration (a), NaCl concentration (b) and hybridization temperature (c) predicted, for each bacterium, through the response surface methodology, using a universal Eubacteria LNA/2’OMe probe (EUB388). Ranges have been stablished assuming a fluorescence intensity of at least 85% of the maximum value. Dash lines represent the conditions selected to be included in initial optimization of LNA/2’OMe-FISH experiments (2 M of urea, 4 M of NaCl and 62 °C of hybridization temperature).

Urea and high salt concentrations seem to be an adequate choice to balance the fluorescence signal among species and to reach a universal hybridization solution. General recommendations for, at least, the starting point on optimization experiments would include approximately 2 M of urea and 4 M of NaCl in hybridization solutions (Fig 6a and 6b). In addition, for 14 bp probes with LNA monomers at every third position of 2′OMe and 64% of GC content, a hybridization temperature of 62°C should be used in initial optimizations (Fig 6c). However, some adjustments will always be necessary for new probes sequences, other sizes or other NAMs.

## Supporting information

S1 Table Experimental levels for the variables (Hybridization temperature, NaCl and formamide) used in the optimization of the LNA/2’OMe-FISH hybridization protocol for *E*. *coli*, *S*. *epidermidis*, *C*. *freundii* and *E*. *faecalis*. (DOCX)

S2 Table Experimental levels for the variables (Hybridization temperature, NaCl and ethylene carbonate) used in the optimization of the LNA/2’OMe-FISH hybridization protocol for *S*. *epidermidis* and *E*. *faecalis*. (DOCX)

S3 Table Experimental levels for the variables (Hybridization temperature, NaCl and urea) used in the optimization of the LNA/2’OMe-FISH hybridization protocol for *P*. *aeruginosa*, *C*. *freundii*, *S*. *epidermidis*. (DOCX)

S4 Table Adjusted quadratic models for the different bacteria in study, in terms of coded values, considering the effect of hybridization temperature (A), denaturant concentration (B) and NaCl concentration (C) in the hybridization solution and their interactions on the predicted fluorescence intensity (a.u). The table also shows the Analysis of variance (ANOVA) for response surface quadratic models obtained for each bacterium and denaturant.
(DOCX)

S1 Fig Surface response plots representing the interactions effect of hybridization temperature and NaCl concentration on the fluorescence response of Gram-negative bacteria (*E*. *coli*, *P*. *aeruginosa* and *C*. *freundii*). The formamide (a), ethylene carbonate (b) and urea (c) concentration was kept constant at the optimum value for each bacterium. Fluorescence values are presented in arbitrary units (a.u).
(DOCX)

S2 Fig Surface response plots representing the interactions effect of hybridization temperature and NaCl concentration on the fluorescence response of Gram-positive bacteria (E. faecalis and S. epidermidis). The formamide (a), ethylene carbonate (b) and urea (c) concentration was kept constant at the optimum value for each bacterium. Fluorescence values are presented in arbitrary units (a.u).
(DOCX)
