# Supplementary material for: Optimizing locked nucleic acid/2’-O-methyl-RNA fluorescence in situ hybridization (LNA/2’OMe-FISH) procedure for bacterial detection
Source: PLoS One. 2019 May 31;14(5):e0217689. doi: 10.1371/journal.pone.0217689 (PMC6544301; doi:10.1371/journal.pone.0217689)
Supplement: S3 Table — (DOCX) [file pone.0217689.s003.docx]

| **Assay** | **Variables** | **Range and level** | | | | |
| --- | --- | --- | --- | --- | --- | --- |
|  |  | -α | -1 | 0 | +1 | +α |
| **2^b^** | x_1_ Hybridization temperature (°C) | 40.0 | 45.1 | 52.5 | 59.9 | 65.0 |
|  | x_2_ [NaCl] (M) | 1.50 | 2.41 | 3.75 | 5.09 | 6.00 |
|  | x_3_ [Urea] (% P/V) | 0.00 | 0.61 | 1.50 | 2.39 | 3.00 |
| **3^c^** | x_1_ Hybridization temperature (°C) | 55.0 | 62.1 | 72.5 | 82.9 | 90.0 |
|  | x_2_ [NaCl] (M) | 1.50 | 2.41 | 3.75 | 5.09 | 6.00 |
|  | x_3_ [Urea] (% P/V) | 0.00 | 0.61 | 1.50 | 2.39 | 3.00 |
| **4^d^** | x_1_ Hybridization temperature (°C) | 40.0 | 47.1 | 57.5 | 67.9 | 75.0 |
|  | x_2_ [NaCl] (M) | 1.50 | 2.41 | 3.75 | 5.09 | 6.00 |
|  | x_3_ [Urea] (% P/V) | 0.00 | 0.61 | 1.50 | 2.39 | 3.00 |

^b^Experimental levels set in the optimization protocol for *P. aeruginosa*.

^c^Experimental levels set in the optimization protocol for *S. epidermidis*.

^d^Experimental levels set in the optimization protocol for *C. freundii*
