# Supplementary material for: Optimizing locked nucleic acid/2’-O-methyl-RNA fluorescence in situ hybridization (LNA/2’OMe-FISH) procedure for bacterial detection
Source: PLoS One. 2019 May 31;14(5):e0217689. doi: 10.1371/journal.pone.0217689 (PMC6544301; doi:10.1371/journal.pone.0217689)
Supplement: S4 Table — The table also shows the Analysis of variance (ANOVA) for response surface quadratic models obtained for each bacterium and denaturant. (DOCX) [file pone.0217689.s004.docx]

| **Denaturant** | **Bacteria** | **Model** | **Model F-value** | **Model *p*-value** | **Model R^2^** |
| --- | --- | --- | --- | --- | --- |
| **Formamide** | *E. coli* | a.u = -1105.36 + 40.62A – 63.07B + 122.13C + 0.29AB -1.34 AC + 4.94BC – 0.32A^2^ – 16.43B^2^ – 24.47C^2^ | 2.20 | 0.1180 | 0.66 |
|  | *P. aeruginosa* | a.u = -1520.49 + 58.58A + 9.56B – 14.01C + 0.27AB + 1.42AC + 2.36BC – 0.51A^2^ – 1.99B^2^ – 14.16C^2^ | 3.82 | 0.0235 | 0.78 |
|  | *C. freundii* | a.u = -283.77 + 8.72A – 2.89B + 75.90C + 0.01AB – 0.39AC – 0.26BC – 0.05A^2^ + 0.01B^2^ – 5.91C^2^ | 3.79 | 0.0248 | 0.77 |
|  | *E. faecallis* | a.u = -901.10 + 33.49A – 2.57B + 119.46C + 0.08AB – 1.37AC – 0.56BC – 0.23A^2^ - 0.00B^2^ + 0.010C^2^ | 6.15 | 0.0045 | 0.85 |
|  | *S. epidermidis* | a.u = -17.50 + 33.49A – 2.04B + 0.52C + 0.04AB + 0.07AC – 0.10BC – 0.02A^2^ - 0.03B^2^ – 0.58C^2^ | 1.12 | 0.4236 | 0.50 |
| **Ethylene carbonate** | *E. coli* | a.u = -973.52 + 39.37A – 26.48B + 65.54C + 0.79AB – 1.27AC + 2.31BC – 0.31A^2^ – 2.18B^2^ – 1.96C^2^ | 14.18 | 0.0001 | 0.93 |
|  | *P. aeruginosa* | a.u = -277.94 + 18.15A – 4.99B – 60.20C + 0.27AB + 0.31AC + 1.12BC – 0.16A^2^ – 0.83B^2^ + 1.35C^2^ | 3.22 | 0.0414 | 0.74 |
|  | *C. freundii* | a.u = -1054.59 + 39.87A – 11.54B + 34.43C + 0.54AB – 0.56AC + 1.18BC – 0.31A^2^ – 1.53B^2^ - 2.42C^2^ | 8.65 | 0.0012 | 0.89 |
|  | *E. faecallis* | a.u = -654.17 + 22.61A + 7.73B + 55.11C + 0.04AB – 0.63AC – 2.69BC – 0.18A^2^ – 0.31B^2^ – 0.67C^2^ | 12.36 | 0.0003 | 0.92 |
|  | *S. epidermidis* | a.u = -674.24 + 16.07A + 37.81B + 79.57C – 0.50AB – 0.23AC + 0.93BC – 0.09A^2^ – 3.74B^2^ – 6.93C^2^ | 3.88 | 0.0230 | 0.78 |
| **Urea** | *E. coli* | a.u = -12.72 + 5.71A + 58.89B + 45.84C – 0.40AB + 0.22AC + 4.36BC - 0.077A^2^ - 8.21B^2^ - 10.64C^2^ | 10.12 | 0.0006 | 0.90 |
|  | *P. aeruginosa* | a.u = -5549.14 + 217.14A + 275.80B + 42.32C – 1.86AB + 0.55AC – 11.88BC – 2.05A^2^ - 38.54B^2^ – 8.19C^2^ | 3.66 | 0.0027 | 0.77 |
|  | *C. freundii* | a.u = -2048.05 + 69.65A + 197.18B + 89.79C – 1.31AB + 0.78AC + 0.48BC – 0.622A^2^ – 44.51B^2^ – 19.61C^2^ | 3.75 | 0.0256 | 0.77 |
|  | *E. faecallis* | a.u = -1314.24 + 37.12A – 99.87B + 264.20C + 1.92AB – 2.02AC + 4.92BC – 0.25A^2^ – 7.08B^2^ – 14.96C^2^ | 20.65 | <0.0001 | 0.95 |
|  | *S. epidermidis* | a.u = -17.50 + 46.38A – 29.35B + 112.38C + 0.07AB + 0.13AC + 2.34BC – 0.32A^2^ – 5.31B^2^ – 12.77C^2^ | 7.60 | 0.0020 | 0.87 |
