# Supplementary figures and images for: Optimizing locked nucleic acid/2’-O-methyl-RNA fluorescence in situ hybridization (LNA/2’OMe-FISH) procedure for bacterial detection
Source: PLoS One. 2019 May 31;14(5):e0217689. doi: 10.1371/journal.pone.0217689 (PMC6544301; doi:10.1371/journal.pone.0217689)

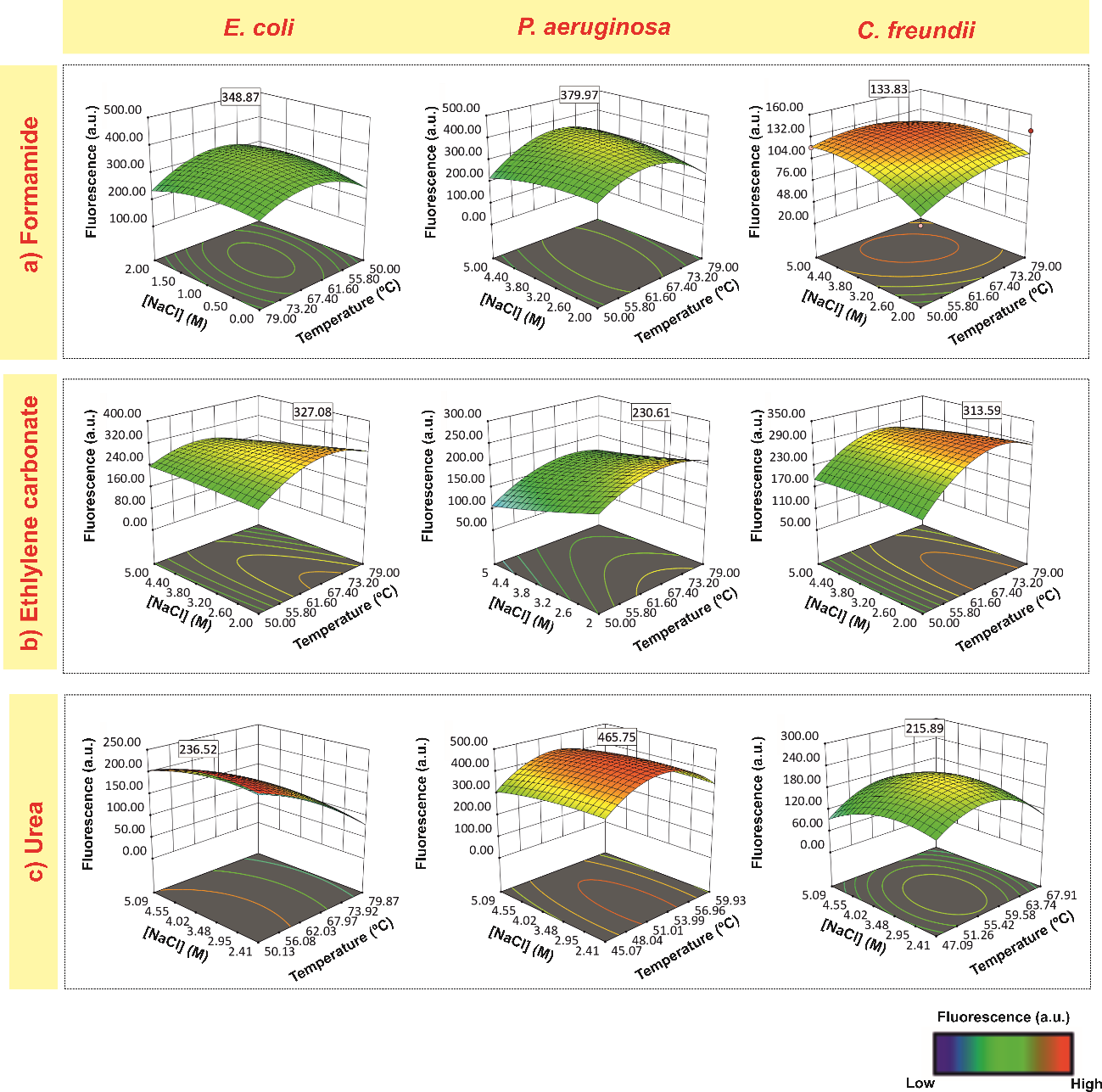

Supplement: S1 Fig — The formamide (a), ethylene carbonate (b) and urea (c) concentration was kept constant at the optimum value for each bacterium. Fluorescence values are presented in arbitrary units (a.u). (DOCX) [file pone.0217689.s005.docx]

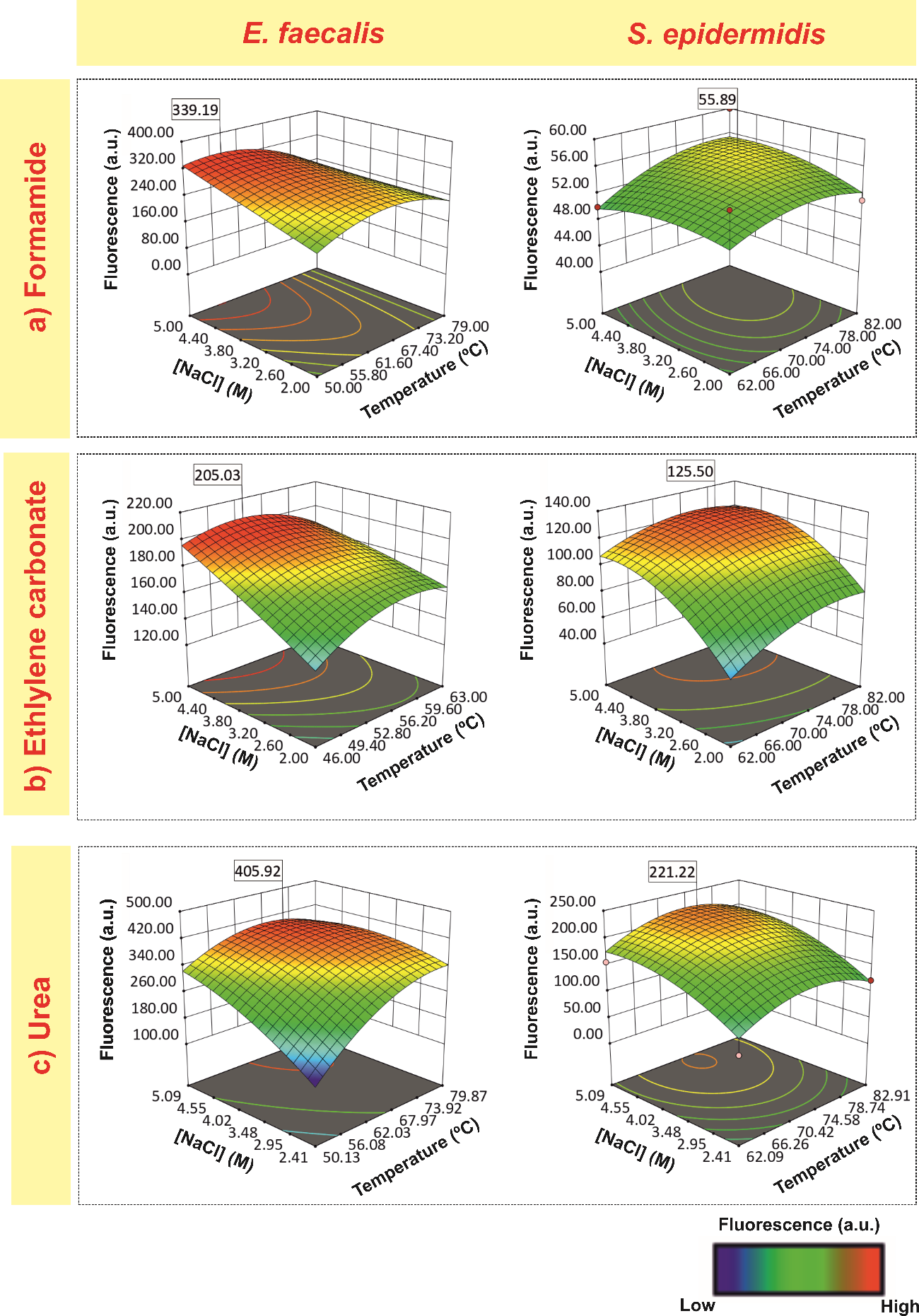

Supplement: S2 Fig — The formamide (a), ethylene carbonate (b) and urea (c) concentration was kept constant at the optimum value for each bacterium. Fluorescence values are presented in arbitrary units (a.u). (DOCX) [file pone.0217689.s006.docx]
